# Supplementary material for: Single Cell Transcriptomic and Chromatin Profiles Suggest Layer Vb Is the Only Layer With Shared Excitatory Cell Types in the Medial and Lateral Entorhinal Cortex
Source: Front Neural Circuits. 2022 Jan 26;15:806154. doi: 10.3389/fncir.2021.806154 (PMC8826650; doi:10.3389/fncir.2021.806154)
Supplement: Supplementary file 14 [file Data_Sheet_1.ZIP › Deep_vs_Sup_Motifs/homerResults/motif5.similar.html]

motif5

## Information for motif5

T
C
G
A
C
T
A
G
T
C
G
A
G
T
C
A
T
C
G
A
C
G
T
A
G
C
A
T
A
C
G
T
A
G
C
T
A
C
G
T
  
Reverse Opposite:  

T
G
C
A
T
C
G
A
T
G
C
A
C
G
T
A
G
C
A
T
A
G
C
T
C
A
G
T
A
G
C
T
G
A
T
C
A
G
C
T
  

|  |  |
| --- | --- |
| p-value: | 1e-51 |
| log p-value: | -1.176e+02 |
| Information Content per bp: | 1.645 |
| Number of Target Sequences with motif | 3394.0 |
| Percentage of Target Sequences with motif | 36.50% |
| Number of Background Sequences with motif | 9518.7 |
| Percentage of Background Sequences with motif | 29.22% |
| Average Position of motif in Targets | 334.8 +/- 245.6bp |
| Average Position of motif in Background | 328.2 +/- 263.3bp |
| Strand Bias (log2 ratio + to - strand density) | 0.0 |
| Multiplicity (# of sites on avg that occur together) | 1.32 |
| Motif File: | file (matrix) reverse opposite |

### Similar de novo motifs found

|  |  |  |  |  |  |  |  |
| --- | --- | --- | --- | --- | --- | --- | --- |
| Rank | Match Score | Redundant Motif | P-value | log P-value | % of Targets | % of Background | Motif file |
| 1 | 0.749 | T G A C G C T A T C G A G T C A C G T A A G C T A G T C G C A T G A C T G C T A C G A T G T A C | 1e-33 | -76.686356 | 8.71% | 5.60% | motif file (matrix) |
| 2 | 0.674 | C G T A C G T A C G T A C G T A A C G T A G T C G T C A A G C T | 1e-32 | -75.494690 | 25.33% | 20.18% | motif file (matrix) |
